# Ecological Interactions of Predatory Mites, *Cheyletus eruditus* (Schrank) (Trombidiformes: Cheyletidae) and *Cheyletus malaccensis* Oudemans, and Prey, *Liposcelis decolor* (Pearman) (Psocodea: Liposcelididae), under Different Thermo-Hygrometric Regimes

**DOI:** 10.3390/insects14090717

**Published:** 2023-08-22

**Authors:** James K. Danso, George P. Opit, Kristopher L. Giles, Bruce H. Noden

**Affiliations:** 1 Department of Entomology and Plant Pathology, Oklahoma State University, 127 Noble Research Center, Stillwater, OK 74078-3033, USA; kris.giles@okstate.edu (K.L.G.); bruce.noden@okstate.edu (B.H.N.); 2 Agricultural Research Station, Fort Valley State University, 1005 State University Drive, Fort Valley, GA 31030-4313, USA

**Keywords:** psocid, biological control, stored-product pest, Cheyletidae, pest management

## Abstract

**Simple Summary:**

Psocids are stored-product pests that are difficult to manage even with the most potent pesticides, including phosphine. Predatory mites, *Cheyletus eruditus* and *Cheyletus malaccensis,* are frequently found associated with pestiferous arthropods in storage environments and can be potential natural enemies for use in stored grain integrated pest management (IPM) systems against psocids. This study aimed to provide quantitative data demonstrating the biological control potential of *C. eruditus* and *C. malaccensis* for managing the psocid species *Liposcelis decolor* in laboratory simulated-storage conditions. Prey population suppression (or percentage prey survival—both refer to extent of decrease (change) in prey population) and progeny replacement efficiency of the predators were assessed under different predator–prey ratios (0:20, 1:20, 2:20, 4:20, and 10:20), temperatures (20, 24, 28, and 32 °C), and relative humidities (RH) (63, 75, and 85%) over 40 days under laboratory conditions of 0:24 (L:D) photoperiod. The results showed that *C. eruditus* and *C. malaccensis* can prey on *L. decolor* to survive, establish, and produce significant numbers of offspring (~96.7–844.4% fold) and caused *L. decolor* population suppression of ~67.1–97.2% for the predator–prey ratios of 1:20, 2:20, 4:20, and 10:20, temperatures of 20, 24, 28, and 32 °C, and RH levels of 63, 75, and 85%. The levels of psocid population suppression achieved indicate the great potential of both predatory mites for psocid management.

**Abstract:**

Predator–prey interactions are linked through trophic relationships, and individual population dynamics are a function of multiple interactions among many ecological factors. The present study considered the efficacy of the predatory mites *Cheyletus eruditus* (Schrank) (Trombidiformes: Cheyletidae) and *Cheyletus malaccensis* Oudemans to manage *Liposcelis decolor* (Pearman) (Psocodea: Liposcelididae). Prey population suppression and progeny replacement efficiency of the predators were assessed under different predator–prey ratios (0:20, 1:20, 2:20, 4:20, and 10:20), temperatures (20, 24, 28, and 32 °C), and relative humidities (RH) (63, 75, and 85%) over 40 days under laboratory conditions of 0:24 (L:D) photoperiod. Suppression of *L. decolor* population when *C. eruditus*-related predator-to-prey ratios of 1:20, 2:20, 4:20, and 10:20 were used was ~61.7, 79.7, 85.1, and 87.5%, respectively, relative to the Control ratio (0:20). In the case of *C. malaccensis*, suppression of 70, 82.1, 92.9, and 96.5%, respectively, was achieved. Although the low 63% RH limited efficacy of these cheyletid mites, both predatory mites caused pest population suppression of ~67.1–97.2% and increased their progeny by ~96.7–844.4% fold for the predator–prey ratios of 1:20, 2:20, 4:20, and 10:20, temperatures of 20, 24, 28, and 32 °C, and RH levels of 63, 75, and 85%. The levels of psocid population suppression achieved indicate the potential of both predatory mites for psocid management.

## 1. Introduction

*Cheyletus eruditus* (Schrank) (Trombidiformes: Cheyletidae) and *Cheyletus malaccensis* Oudemans are the most dominant and widely distributed cheyletid species in post-harvest agricultural systems in temperate and tropical regions [1,2,3]. Both predatory mites are natural enemies of multiple arthropods and prey on mite and non-mite pests of stored products [4,5,6], including psocids (Psocodea: Liposcelididae) [7]. Predator–prey interactions are linked through trophic relationships, and individual population dynamics are a function of multiple interactions among many biotic and abiotic variables [8,9,10]. For example, a natural enemy complex may exist in the storage community due to the presence of different prey species and leads to trophic interactions such as competition, interference, cannibalism, and intraguild predation even among conspecifics with a profound effect on biocontrol agents in pest management programs [11,12,13,14]. Prey biotic-related factors such as prey characteristics, prey type and composition, seasonal occurrence, and spatial abundance are major determinants for the establishment, colonization, predation, survivorship, and reproduction of predators to allow them to effectively manage pest populations [5,15,16,17,18,19]. Similarly, physical conditions within storage environments including temperature and relative humidity (RH) are key variables that influence the overall outcome of ecological interactions; predators and prey can survive and thrive under different temperature and RH ranges favorable for their growth and development. For instance, *C. malaccensis* can reproduce at temperatures between 17.5 and 35 °C and develop in the range between 11.6 and 37.8 °C [5,10,14]. *Cheyletus eruditus* populations multiply rapidly during summer and autumn when temperatures are high, whereas its prey *Acarus siro* L. (Sarcoptiformes: Acaridae) population levels decline substantially as a result of extreme temperatures and RH [20]. *Cheyletus eruditus* can complete its life cycle at temperatures and RH ranging from 12 to 35 °C and 60 to 90%, respectively [21].

Release ratio (predator-to-prey ratio) is a prerequisite variable to establish prior to the release of biocontrol agents, and this is critical for successful biological control programs [9]. The degree of control would be influenced by how many predators are released at a given pest infestation level. Thus, pest control would be poor if a limited number of predators were released, and, on the other hand, the release of predators at a higher density would likely lead to underutilization of their control potential through density-dependent negative feedbacks [14]. To date, little is known about the potential of the cheyletid mites *C. eruditus* and *C. malaccensis* to manage psocid populations under physical conditions that exist in storage environments. Currently, only [7] has documented the behavioral and ecological aspects of *C. eruditus* and how these relate to its potential to suppress *Liposcelis decolor* (Pearman) (Psocodea: Liposcelididae) populations in stored grain. *Liposcelis decolor* is considered as one of the most insecticide-tolerant stored-product insect pests and is difficult to manage even with the most potent pesticides. For example, susceptible laboratory strains of *L. decolor* can tolerate higher concentrations of phosphine up to 249.76 ppm [22]. Previous data [6,23] on the foraging behaviors of *C. eruditus* and *C. malaccensis* showed that these cheyletids had the potential to suppress *L. decolor* population under the same temperature (24 ± 1 °C) and RH (85 ± 5%) conditions. Given that the general performance of an effective biocontrol agent is a function of multiple ecological interactions among many biotic and abiotic variables, the evaluation of predator–prey interaction based on other variables such as predatory-to-prey ratio and thermo-hygrometric factors is necessary to investigate. The current study aimed to assess the ecological interaction between the cheyletid mites *C. eruditus* and *C. malaccensis* and the psocid species *L. decolor*. How *L. decolor* population dynamics is influenced by different predator-to-prey ratios at varying temperatures and RH regimes was investigated. Specifically, percentage prey suppression (suppression efficiency) and progeny replacement of *C. eruditus* and *C. malaccensis* were estimated and compared under different predator-to-prey ratios and thermo-hygrometric conditions (temperature and RH). This study will provide the baseline information for further evaluation of these cheyletids to facilitate their incorporation into existing IPM systems for managing psocids.

## 2. Materials and Methods

### 2.1. Mites Colonies

The source and rearing of laboratory colonies of *A. siro* were as described in [6,23].

### 2.2. Rearing of Predators, Cheyletus eruditus and Cheyletus malaccensis

Rearing methods for *C. eruditus* and *C. malaccensis* were as described in [6,23]. These methods are modified from a mass rearing protocol for *C. eruditus* as described by [24]. Only adult female (♀) predatory mites were used for this study and were selected based on what is described in [6,21,23,25].

### 2.3. Rearing of Prey, Liposcelis decolor

*Liposcelis decolor*, used as prey, was reared according to methods in [6,23,26].

### 2.4. Experimental Arenas

Experimental arenas consisted of two 6.0 cm diameter Petri dishes (forming a total cylindrical surface area of 113.04 cm^2^; the total migration area of the individual predator in a closed cylinder) (60 × 15 mm, Style Polystyrene, Becton Dickinson and Company, Franklin Lakes, NJ, USA). Other aspects of the arenas were as described in [6,23]. The total migration area of individual prey was 47.12 cm^2^. The basal Petri dishes contained 5.0 g of cracked wheat covering the entire bottom portion of each arena, which was food for *L. decolor*.

### 2.5. Predation and Progeny of Cheyletus eruditus and Cheyletus malaccensis

**Figure 1:**
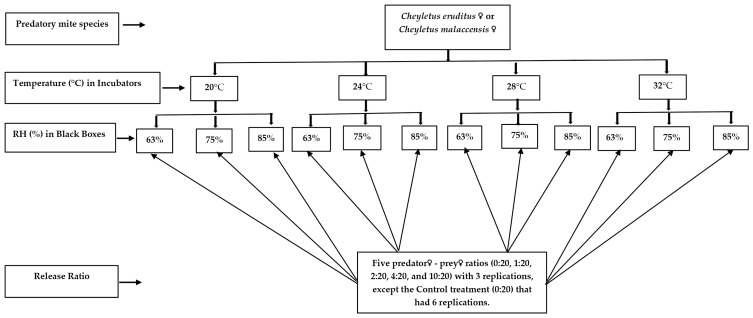
A flowchart of experimental design for ecological interactions of the predator species *C. eruditus* or *C. malaccensis* over a 40 day period. Predatory mite species were *Cheyletus eruditus* or *C. malaccensis*, initial prey density was 20 females (♀) of *L. decolor*, and there were 5 levels of predator-to-prey (release) ratio (0:20, 1:20, 2:20, 4:20, and 10:20), 4 levels of temperature (°C) (20, 24, 28, and 32 °C), and 3 levels of relative humidity (RH) (63, 75, and 85%).

Prey (adult female *L. decolor*) population suppression levels by *C. eruditus* or *C. malaccensis* in 5.0 g grain samples per 113.04 cm^2^ experimental arenas were considered at different predator-to-prey ratios under varying temperatures and RH over a 40 day experimental period. Predator-to-prey ratios of 0:20, 1:20, 2:20, 4:20, and 10:20 were assigned to well-labeled experimental arenas. Adult females freshly molted from tritonymph (3–7 days old) were selected from pure cultures of *C. eruditus* or *C. malaccensis* and were assigned separately to the experimental arenas. Twenty (20) female adults (♀) of *L. decolor* were introduced into each arena, however, with different predatory mite numbers (0, 1, 2, 4, or 10), with zero (0) or a predator-to-prey ratio of 0:20 being the Control treatment. Predatory mites were starved for 24 h prior to introduction to their prey, as this reduces initial variability in oviposition, standardizes hunger level, and initiates a nomadic period [7,27,28]. The predator-inoculated experimental arenas together with the Control treatments were randomly arranged in plastic boxes (18 × 15 × 12 cm) painted black, which had either NaNO_2_ (Sodium nitrite, anhydrous, free-flowing, Redi-Dri^TM^, ACS reagent, ≥97%, 799416-2.5KG, Sigma-Aldrich, Inc., St. Louis, MO, USA), NaCl, or KCl saturated solution beneath perforated false floors to maintain 63, 75, or 85% RH, respectively, and these were placed inside growth chambers maintained at 20, 24, 28, or 32 °C for 40 days. The experimental design was a three-factor factorial Completely Randomized Design. Factors were predatory–prey ratio with five levels (0:20, 1:20, 2:20, 4:20, and 10:20), temperature with four levels (20, 24, 28, and 32 °C), and RH with three levels (63, 75, and 85%); hence, a 5 × 4 × 3 factorial CRD was used. Altogether, there were 60 treatments (factor level combinations) for either *C. eruditus* or *C. malaccensis*. Each treatment was replicated three times. Arenas were accordingly set up in the respective relative humidity boxes in each incubator maintained at each of the temperatures investigated. Separate RH boxes were assigned to each predatory mite species to prevent cross-infestation of predatory mites. Each growth chamber (temperature level) had all the combined levels of release ratio and RH for both predatory mites. The treatment replicates were run concurrently for both predatory mite species to avoid temporal variation among response variables (Figure 1). After 40 days, the number of surviving adults and nymphs of *L. decolor* in each treatment were counted to assess and compare prey suppression efficiency of *C. eruditus* or *C. malaccensis* under the different predator-to-prey ratios, temperatures, and RH. This was determined by comparing treatments with predators (1, 2, 4, and 10 density of a predatory mite species) against the Control treatment (only prey; no predator) under different temperatures (20, 24, 28, and 32 °C) and RH (63, 75, and 85%) after 40 days. Prey population suppression or percentage prey survival both refer to extent of decrease (change) in prey population. Additionally, the reproductive responses of each predatory mite species were estimated by counting all the mobile stages of each predator species and using the data to estimate the per capita progeny production of *C. eruditus* or *C. malaccensis* under the different predator-to-prey ratios, temperatures, and RH.

### 2.6. Statistical Analysis

The mean percentage prey survival (%) and per capita progeny production (%) of *C. eruditus* and *C. malaccensis* were estimated using the percentage change model (Furey 2019): [(N − N_o_/N_o_)*100)]. In this model, N_o_ and N represent the initial and final predator or prey numbers, respectively. The estimated percentage prey survival and progeny production were compared across the five predator–prey ratios (0:20, 1:20, 2:20, 4:20, and 10:20), four temperatures (20, 24, 28, and 32 °C), and three RH (63, 75, and 85%) using generalized linear mixed model methods for each of the predator species. PROC GLIMMIX modeled the fixed effects of predator–prey ratio, temperature, and RH and interactions for each of the response variables with the specified response distribution (~Gaussian) in SAS. Data were analyzed using a square root transformation and a heterogeneous variances model since the response variables exhibited heterogeneity of variances. Least squares means were compared for the appropriate significant effects using the Tukey method. All tests were conducted at the nominal 0.05 level of significance. Means and standard errors for each factor combination are reported. All data were analyzed using SAS/STAT software Version 9.4 (SAS Institute, Cary, NC, USA).

## 3. Results

### 3.1. Effects of Predator-to-Prey Ratio, Temperature, and RH on Percentage Survival of L. decolor

The results of percentage prey survival after 40 days of exposure of *L. decolor* to predatory mites showed that the three-way interaction of predator-to-prey ratio (hereafter referred to as release ratio), temperature, and relative humidity (RH) was significant (*p* < 0.05) for *C. eruditus* (Table 1). Percentage *L. decolor* survival increased with increasing temperature along with decreasing release ratio, however, prey numbers increased with RH and then declined considerably, especially at the highest temperature and RH (32 °C and 85%) under all release ratios (Table 2). Relative to the Control treatment (0:20), *C. eruditus* substantially suppressed *L. decolor* population size by ~87.5%, 85.1%, 79.7%, and 61.7% in the 10:20, 4:20, 2:20, and 1:20 release ratios, respectively, under the combined temperature and RH effects (Table 2). For *C. malaccensis*, the three-way interaction of release ratio, temperature, and relative humidity (RH) was not significant (*p* > 0.05) (Table 1). However, all the two-way interactions between release ratio, temperature, or RH were significant (*p* < 0.05) (Table 1) except for temperature and RH. At 10:20 release ratio, near complete or complete prey suppression (~100%) was achieved by *C. malaccensis* under temperature and RH ranges of 28–32 °C and 63–75%, respectively (Table 2). Relative to the Control treatment, *C. malaccensis* substantially suppressed *L. decolor* population size by ~96.5%, 92.9%, 82.1%, and 70.0% in the 10:20, 4:20, 2:20, and 1:20 release ratios, respectively, under the combined temperature and RH effects (Table 2).

### 3.2. Effect of Predator-to-Prey Ratio, Temperature, and RH on C. eruditus and C. malaccensis Progeny Production

In relation to per capita predator progeny production (percentage increase in predator’s population after 40 days), the interaction of release ratio, temperature, and RH was significant (*p* < 0.05) for both predatory mites, *C. eruditus* and *C. malaccensis* (Table 1). For *C. eruditus*, the maximum offspring production (excluding the egg stage) was observed at 24 °C and 75 and 85% RH across all the tested release ratios (~175.0–2800.0% increase in population per predator) (Table 3) but was substantially lower under conditions of 28 °C and 63% RH (~0.0% offspring per predator) and 32 °C and 63% RH (≤16.7% increase in offspring per predator) across all the release ratios. Thus, a general trend of increase in progeny production at the low release ratios of 1:20 and 2:20, temperatures of 20 and 24 °C, and RH of 75 and 85% was observed in *C. eruditus* (Table 3). Similarly, for *C. malaccensis*, the highest progeny production was observed at 24 °C and 75 and 85% RH across all the tested release ratios (~196.7–1500.0% increase in population per predator) (Table 3). However, no offspring of *C. malaccensis* were produced under conditions of 28 and 32 °C and 63% RH across all the tested release ratios. Under conditions of 24 °C and 63% RH; offspring were produced only at the release ratio of 10:20 (Table 3). Mostly, a trend of increase in progeny production at 75 and 85% RH and at temperatures of 20 and 24 °C in all release ratios was found in the case of *C. malaccensis* (Table 3).

## 4. Discussion

The most significant result of this study was that *C. eruditus* and *C. malaccensis* were found to effectively prey on *L. decolor* and suppress its population in a wide range of ecological conditions. Our data have confirmed observations by several reports that both predatory mites are good potential natural enemies of most pestiferous insects in stored products, including psocids in the genera *Liposcelis* and *Lepinotus* [6,7,23,29,30,31,32,33]. In the Czech Republic, for example, *C. eruditus* (Cheyletin^®^) is the only commercialized predatory mite approved for use to manage mite pests in food storage systems such as in a stored-grain mass and in grain residues, debris in empty stores, or seed stores in [1,34]. Therefore, these predatory mites can be used for disinfesting empty storehouses (warehouses), storehouses with bagged commodities, pallets, and transportation containers of psocids through augmentation by inundative release to minimize pesticide used. This study has also showed that *C. eruditus* and *C. malaccensis* require a small number of *L. decolor* to complete their development and can parthenogenetically augment their offspring in grain under a wide range of release ratios (1:20, 2:20, 4:20, and 10:20 predator-to-prey ratios) while adopting cannibalism as a means of survival when *L. decolor* are absent (as shown by the data). Schöller et al. [21] reported similar biological characteristics in the warehouse pirate bug *Xylocoris flavipes* (Reuter) (Hemiptera: Anthocoridae), one of the most-studied and efficient stored-grain predatory insects registered by the Environmental Protection Agency (EPA) for use against stored-product insect pests in the United States [35].

The current study revealed that *C. eruditus* and *C. malaccensis* are prolific under different release ratios in a diverse range of temperatures and relative humidities when fed on *L. decolor*. The rapid growth rate of prey at 32 °C and 75% RH may have contributed to the higher survival rate in the presence of either *C. eruditus* or *C. malaccensis* at varying predator densities. However, the optimal prey suppression capacities and progeny production of both predators were mostly found in dissimilar biotic and abiotic conditions. For instance, previous research by Kucerova [7] showed that *C. eruditus* prey on all developmental stages of *L. decolor* and significantly suppressed the prey population size in grain samples under laboratory conditions of 25 °C and 85% RH and in 40 days of exposure. In that study, it was found that at 1:2 release ratio, the number of individual *L. decolor* decreased substantially from ~100 individuals in the Control treatment to ~20 individuals in the predator treatments, representing ~80.0% population suppression; in the 1:5 released ratio, the population size decreased considerably from ~190 to ~30 individuals of *L. decolor*, representing ~84.2% population suppression. This range of *L. decolor* population suppression was consistent with the results of the current study, although a trend of increased prey mortality at the higher release ratios was found in this study. Thus, *C. eruditus* substantially suppressed *L. decolor* population by 61.7–87.5% in 1:20, 2:20, 4:20, and 10:20 release ratios, whereas that of *C. malaccensis* was mostly higher with the estimated values of ~70.0–96.5% in the 1:20, 2:20, 4:20, and 10:20 release ratios when compared with the Control population. A similar range of prey mortalities was reported when 13 natural enemies of 19 stored-products insect pests were assessed; a range of 70.0–100% prey suppression efficiency was reported [35]. 

Based on the present study, it can be deduced that *L. decolor* mortality by *C. eruditus* and *C. malaccensis* would be low if a limited number of predators are released for biological control. However, predators released at higher numbers would under-perform due to density-dependent factors such as competition, mutual interference, and cannibalism [14]. Therefore, establishing predator–prey balance either spatially or temporally through accurate estimation of release ratio would be important for a successful biological control program. Again, this information is critical for commercial production of *C. eruditus* and *C. malaccensis*, where a lower release ratio is recommended for mass rearing and psocid management. The exact critical *L. decolor* population level (density) that limits successful prey suppression by both predators was not established in the present study due to significant prey mortality by predators observed in all the release ratios (1:20, 2:20, 4:20, and 10:20 predator–prey ratios). However, it is expected that *C. eruditus* and *C. malaccensis* would effectively manage *L. decolor* populations even when the release ratio is as low as 1:20, and in temperatures of 20, 24, and 28 °C, and 75% RH (RH that is optimal for the growth and developmental of *L. decolor*). Both predators increased their progeny production with decreasing release ratio. Therefore, for inoculative release of *C. eruditus*, lower release ratios (≤1:10 predator–prey ratios) should be targeted where the predator’s population can increase considerably to ~1416.7–2800.0% under a temperature of 24 °C and 75 and 85% RH after 40 days of release. Likewise, with lower release ratios (1:20 and 2:20) and temperature and RH conditions of 24 °C and 75 and 85%, *C. malaccensis* population could increase substantially by ~500.0–1500.0% after 40 days when managing psocid infestations. Based on data from this study, RH of ≤63% in the storage environment or rearing facilities could hinder the growth, development, and proliferation of both predatory mites, especially when the exposure temperature is 28 °C or higher. However, temperature ranges of 27–28 °C were reported by [5,10] as the optimal temperature for growth and development of *C. malaccensis*. 

Ecological factors in storage facilities influence the overall performance of biological control agents [21]. The contradictory trend between the current results and the work by [7] and the higher prey mortality in the present findings can probably be attributed to the influence of a wider range of temperature (20, 24, 28, and 32 °C) and RH (63, 75, and 85%) used in the present work. Athanassiou et al. [36] noted that the ecology of *C. malaccensis* in various commodities and in different types of storage facilities was mostly influenced by abiotic factors, such as temperature and moisture, and not by predation. The variation in *C. eruditus* efficiency can be related to the existence of various biotypes of *Cheyletus* spp. [3]. The marginal differences in performances of *C. malaccensis* over *C. eruditus* in most of the release ratios may be explained by the texture of the medium used in the experimental arenas (coarse-wheat grain), which is mostly preferred by *C. malaccensis* and not *C. eruditus*. Hubert et al. [37] reported that *C. eruditus* is more common in grain residues, while *C. malaccensis* is mostly found in grain mass because of its ability to penetrate bulk grain. Despite their co-existence in the storage environment [36,38], it is generally considered that *C. eruditus* is more adapted in tropical conditions, while *C. malaccensis* is more abundant in temperate regions [3]. The present study showed a general trend of increasing progeny production with decrease in release ratio and temperature and increase in RH for both predatory mites. *Cheyletus eruditus* and *C. malaccensis* increased their population size considerably at the release ratio of 1:20, temperatures of 20 °C and 24 °C, and 75% and 85% RH levels. However, 63% RH proved detrimental to predator survival and population growth across all the release ratios tested. This implies that RH would be the main limiting factor that can influence the level of control achieved by *C. eruditus* and *C. malaccensis* in any biological control program against psocid species, especially *Liposcelis* species such as *L. obscura* that can surprisingly survive in RH conditions close to 63% [39]. Therefore, effective inundative release of these cheyletid mites should target periods when ambient or storage RH is >63% in order to enable the proliferation of *C. eruditus* and *C. malaccensis* to enhance their prey suppression efficiency [14,20,21].

The current study has for the first time provided information on the efficacy of cheyletid mites *C. eruditus* and *C. malaccensis* to manage psocids (*L. decolor*) in diverse thermo-hygrometric regimes under laboratory stimulations. The capacity of both predatory mites to effectively suppress *L. decolor* populations was established, while the progeny production by predators was significant under the tested biotic and abiotic conditions. Although the low 63% RH limited efficacy of these cheyletid mites, both predatory mites caused population suppression of ~61.7–96.5% and increased their progeny by ~96.7–844.4% for the 1:20, 2:20, 4:20, and 10:20 release ratios, temperatures of 20, 24, 28, and 32 °C, and 75% and 85% RH levels after 40 days of exposure to psocids infesting grain. These temperature and RH ranges represent physical conditions that permit survival of psocids. The levels of psocid population suppression achieved indicate the good potential of both predatory mites for psocid management. Whereas laboratory assessment is a critical step along a continuum of screening and evaluation procedures for the selection of efficient biocontrol agents, laboratory simulations alone do not allow predictions of the success of predatory mites under the field conditions. Therefore, further assessment under storage ecological conditions (field trials) is needed. This should include wider release ratios, simultaneous interactions of predators, prey preference, cannibalism, and life table parameters of predators. Moreover, research on the compatibility of these cheyletids mites with other stored-product pest management strategies (especially the effects of residual pesticides on survival of these mites) should be conducted to enable integration of these cheyletids in storage IPM systems for psocid pest management in the United States.

## Figures and Tables

**Table 1:** Summary of the tests for the fixed effects of predator–prey ratio (P-P-R), temperature (T), and relative humidity (RH) and interactions for percentage prey surviving (%) and per capita progeny production (% increase in population) of predatory mites for *Cheyletus eruditus* (CE) or *Cheyletus malaccensis* (CM) exposed to initial prey density of 20 females of *L. decolor* over a 40 day period. The symbol (*) denotes interaction between factors.

Variable	Predator	Source	DF	*F*	*p* Value
Prey survival	CE	T	3, 91.64	6.10	0.0008
		RH	2, 91.64	30.90	<0.0001
		T*RH	6, 91.64	5.41	<0.0001
		P-P-R	4, 57.25	249.09	<0.0001
		T*P-P-R	12, 80.62	7.07	<0.0001
		RH*P-P-R	8, 73.59	6.68	<0.0001
		T*RH*P-P-R	24, 87.52	1.81	0.0250
	CM	T	3, 84.59	0.42	0.7383
		RH	2, 84.59	25.37	<0.0001
		T*RH	6, 84.59	1.61	0.1539
		P-P-R	4, 60.7	469.66	<0.0001
		T*P-P-R	12, 80.7	12.13	<0.0001
		RH*P-P-R	8, 73.89	7.29	<0.0001
		T*RH*P-P-R	24, 87.18	1.62	0.0539
Predator progeny	CE	T	3, 66.28	39.08	<0.0001
		RH	2, 66.28	247.13	<0.0001
		T*RH	6, 66.28	8.65	<0.0001
		P-P-R	3, 49.45	43.95	<0.0001
		T*P-P-R	9, 62.3	6.02	<0.0001
		RH*P-P-R	6, 57.99	13.76	<0.0001
		T*RH*P-P-R	18, 66.04	3.98	<0.0001
	CM	T	3, 58.69	63.85	<0.0001
		RH	2, 58.69	137.73	<0.0001
		T*RH	6, 58.69	8.20	<0.0001
		P-P-R	3, 46.72	10.35	<0.0001
		T*P-P-R	9, 58.59	3.02	0.0050
		RH*P-P-R	6, 54.64	17.91	<0.0001
		T*RH*P-P-R	18, 61.77	4.53	<0.0001

**Table 2:** Mean percentage prey surviving (±SE) over a 40 day period. Predatory mite species (P) were *Cheyletus eruditus* (CE) or *C. malaccensis* (CM), initial prey density was 20 females of *L. decolor*, and there were 5 levels of predator-to-prey ratio (P-P-R) (0:20, 1:20, 2:20, 4:20, and 10:20), 4 levels of temperature (T) (20, 24, 28, and 32°), and 3 levels of relative humidity (RH) (63, 75, and 85%).

P	T	RH	P-P-R				
			0:20	1:20	2:20	4:20	10:20
CE	20	63	35.8 ± 5.23 aH	21.7 ± 3.33 bDE	10.0 ± 5.77 bC	10.0 ± 2.89 bAB	1.7 ± 1.67 cD
	20	75	59.2 ± 4.90 aDE	33.3 ± 3.33 bB	16.7 ± 8.33 cBC	8.3 ± 4.41 dBC	5.0 ± 2.89 eBC
	20	85	74.2 ± 9.08 aCD	21.7 ± 3.33 bDE	13.3 ± 4.41 bBC	8.3 ± 4.41 cBC	1.7 ± 1.67 dD
	24	63	42.5 ± 2.14 aGH	21.7 ± 6.01 bDE	10.0 ± 5.00 cC	6.7 ± 4.41 dCD	6.7 ± 1.67 dBC
	24	75	63.3 ± 8.82 aDE	25.0 ± 2.89 bCD	13.3 ± 1.67 cBC	6.7 ± 1.67 cB	3.3 ± 1.67 dCD
	24	85	65.8 ± 9.87 aDE	20.0 ± 0.00 bD	13.3 ± 6.01 bcBC	5.0 ± 5.00 dD	6.7 ± 1.67 cB
	28	63	44.0 ± 3.87 aFG	23.3 ± 3.33 bD	13.3 ± 1.67 bBC	5.0 ± 2.89 cD	5.0 ± 5.00 cBC
	28	75	207.5 ± 21.16 aA	51.7 ± 7.26 bA	11.7 ± 1.67 cBC	8.3 ± 1.67 cBC	8.3 ± 1.67 cAB
	28	85	95.8 ± 7.90 aBC	33.3 ± 6.01 bB	16.7 ± 1.67 cA	5.0 ± 5.00 dBC	3.3 ± 3.33 eCD
	32	63	70.8 ± 10.60 aDE	16. 7 ± 1.67 bE	3.3 ± 1.67 cD	1.7 ± 1.67 dD	1.7 ± 1.67 dD
	32	75	234.2 ± 21.07 aA	58.3 ± 9.28 bA	16.7 ± 4.41 cA	13.3 ± 4.41 cA	10.0 ± 0.00 cA
	32	85	110.8 ± 8.60 aB	38.3 ± 4.41 bB	10.0 ± 5.00 cC	5.0 ± 5.00 dD	1.7 ± 1.67 dD
							
CM	20	63	44.2 ± 5.69 aG	18.3 ± 3.33 bC	11.7 ± 6.01 cC	6.7 ± 3.33 cBC	8.3 ± 3.33 cAB
	20	75	64.2 ± 5.97 aF	35.0 ± 5.00 bA	21.7 ± 3.33 bA	10.0 ± 2.89 cA	5.00 ± 2.89 dBC
	20	85	73.3 ± 5.87 aEF	33.3 ± 1.67 bA	28.3 ± 10.14 bA	8.3 ± 3.33 cAB	6.7 ± 1.67 cAB
	24	63	58.3 ± 4.41 aFG	28.3 ± 3.33 bAB	15.0 ± 2.89 cBC	6.7 ± 1.67 dAB	1.7 ± 1.67 dC
	24	75	95.0 ± 8.06 aDE	28.3 ± 6.01 bAB	23.3 ± 6.67 bA	10.0 ± 5.77 cAB	1.7 ± 1.67 dC
	24	85	90.8 ± 7.35 aDE	36.7 ± 3.33 bA	28.3 ± 4.41 bA	10.0 ± 5.77 cAB	1.7 ± 1.67 dC
	28	63	60.0 ± 4.08 aFG	16.7 ± 4.41 bC	5.0 ± 5.00 cD	1.7 ± 1.67 cD	0.0 ± 0.00 dD
	28	75	165.8 ± 12.07 aB	38.3 ± 6.01 bA	20.0 ± 5.00 bAB	5.0 ± 5.00 cC	0.0 ± 0.00 dD
	28	85	115.0 ± 12.78 aCD	38.3 ± 8.82 bA	26.7 ± 4.41 bA	8.3 ± 3.33 cAB	1.7 ± 1.67 dC
	32	63	75.8 ± 4.36 aEF	16.7 ± 4.41 bC	10.0 ± 2.89 bC	5.0 ± 5.00 cC	0.0 ± 0.00 dD
	32	75	220.8 ± 15.24 aA	38.3 ± 3.33 bA	13.3 ± 10.91 cC	5.00 ± 5.00 dC	3.3 ± 1.67 dBC
	32	85	135.0 ± 13.10 aC	30.0 ± 8.66 bAB	10.0 ± 5.77 cC	6.7 ± 3.33 dBC	10.0 ± 2.896 cdA

Significant differences among P-P-R for each T*RH interaction are denoted with different lower-case letters (within the same row) for each predator, and differences among T*RH interaction for each P-P-R are denoted by different upper-case letters for each predator (within a column) under a given variable (*p* < 0.05, LSMeans under Proc GLIMMIX in SAS).

**Table 3:** Mean per capita progeny production (%) (±SE) over a 40 day period. Predatory mite species (P) were *C. eruditus* (CE) or *C. malaccensis* (CM), initial prey density was 20 females of *L. decolor*, and there were 5 levels of predator-to-prey ratio (P-P-R) (1:20, 2:20, 4:20, and 10:20), 4 levels of temperature (T) (20, 24, 28, and 32°), and 3 levels of relative humidity (RH) (63, 75, and 85%).

P	T	RH	P-P-R			
			1:20	2:20	4:20	10:20
CE	20	63	266.7 ± 120.19 aE	66.7 ± 16.67 bE	25.0 ± 14.43 bD	53.3 ± 14.53 bD
	20	75	1366.7 ± 375.65 aBC	816.7 ± 142.40 bB	333.3 ± 22.05 cBC	326.7 ± 48.07 cAB
	20	85	1200.0 ± 321.46 aBC	766.7 ± 92.80 bB	308.3 ± 16.7 cBC	133.3 ± 14.53 dCD
	24	63	33.3 ± 33.33 aF	16.7 ± 16.67 aE	50.0 ± 28.87 aD	36.7 ± 8.82 aD
	24	75	1233.3 ± 392.99 aBC	1416.7 ± 365.53 aA	1066.7 ± 130.97 aA	190.0 ± 60.83 bBC
	24	85	2800.0 ± 763.76 aA	1733.3 ± 337.06 bA	775.0 ± 202.07 cA	316.7 ± 76.23 dAB
	28	63	0.0 ± 0.00 aF	0.0 ± 0.00 aE	0.0 ± 0.00 aD	0.0 ± 0.00 aD
	28	75	1100.0 ± 264.58 aBC	433.3 ± 60.09 bC	208.3 ± 50.69 cC	146.7 ± 37.12 cCD
	28	85	366.7 ± 66.67 aDE	366.7 ± 72.65 aCD	250.0 ± 50.00 aBC	116.7 ± 17.64 bCD
	32	63	0.0 ± 0.00 aF	0.0 ± 0.00 aE	0.0 ± 0.00 aD	16.7 ± 16.67 aD
	32	75	500.0 ± 173.21 aCD	116.7 ± 72.65 bD	116.7 ± 104.42 bCD	386.7 ± 108.37 aA
	32	85	1266.7 ± 218.58 aBC	516.7 ± 120.19 bB	375.0 ± 90.14 bB	143.3 ± 31.80 cCD
						
CM	20	63	33.3 ± 33.33 cE	233.3 ± 130.17 aC	16.7 ± 16.67 cCD	116.7 ± 14.53 bBC
	20	75	533.3 ± 145.30 aBC	283.3 ± 33.33 abB	241.7 ± 44.10 bcB	180.0 ± 17.32 cAB
	20	85	766.7 ± 317.98 aB	666.7 ± 158.99 abAB	341.7 ± 65.09 bAB	150.0 ± 36.06 cAB
	24	63	0.0 ± 00.00 bF	0.0 ± 0.00 bE	0.0 ± 0.00 bD	83.3 ± 23.33 aC
	24	75	400.0 ± 173.21 aBC	500.0 ± 144.34 aAB	416.7 ± 65.09 aA	196.7 ± 23.33 bAB
	24	85	1500.0 ± 416.33 aA	783.3 ± 109.29 bA	575.00 ± 86.60 bA	200.0 ± 45.83 cA
	28	63	0.0 ± 0.00 aF	0.0 ± 0.00 aE	0.0 ± 0.00 aD	0.0 ± 0.00 aD
	28	75	33.3 ± 33.33 bE	33.3 ± 33.33 bD	50.0 ± 25.00 bC	120.0 ± 17.32 aBC
	28	85	500.0 ± 152.75 aBC	200.0 ± 125.83 bC	166.7 ± 33.33 bBC	73.3 ± 18.56 cCD
	32	63	0.0 ± 0.00 aF	0.0 ± 0.00 aE	0.0 ± 0.00 aD	0.0 ± 0.00 aD
	32	75	200.0 ± 115.47 aCD	0.0 ± 0.00 cE	16.7 ± 16.67 bC	23.3 ± 8.82 bCD
	32	85	666.7 ± 145.30 aBC	16.7 ± 16.67 bE	0.0 ± 0.00 cD	16.7 ± 12.02 bCD

Significant differences among P-P-R for each T*RH interaction are denoted with different lower-case letters (within the same row) for each predator, and differences among T*RH interaction for each P-P-R are denoted by different upper-case letters for each predator (within a column) under a given variable (*p* < 0.05, LSMeans under Proc GLIMMIX in SAS).

## Data Availability

Data is contained within the article.
